# Intestinal immunity in hypopituitary dwarf mice: effects of age

**DOI:** 10.18632/aging.101393

**Published:** 2018-03-02

**Authors:** Xin Wang, Justin Darcy, Chuan Cai, Junfei Jin, Andrzej Bartke, Deliang Cao

**Affiliations:** 1 Department of Medical Microbiology, Immunology, and Cell Biology, Southern Illinois University, School of Medicine, Springfield, IL 62702, USA; 2 Department of Internal Medicine, Southern Illinois University, School of Medicine, Springfield, IL 62702, USA; 3 Division of Stem Cell Regulation and Application, State Key Laboratory of Chinese Medicine Powder and Medicine Innovation in Hunan (incubation), Hunan University of Chinese Medicine, Changsha, Hunan 410208, China; 4 China-USA Lipids in Health and Disease Research Center, Guilin Medical University, Guilin 541001, Guangxi, China

**Keywords:** aging, dwarfism, colonic development, intestinal immunity, immune cells

## Abstract

Hypopituitary dwarf mice demonstrate advantages of longevity, but little is known of their colon development and intestinal immunity. Herein we found that Ames dwarf mice have shorter colon and colonic crypts, but larger ratio of mesenteric lymph nodes (MLNs) over body weight than age-matched wild type (WT) mice. In the colonic lamina propria (cLP) of juvenile Ames mice, more inflammatory neutrophils (Ā: 0.15% vs. 0.03% in WT mice) and monocytes (Ā: 7.97% vs. 5.15%) infiltrated, and antigen presenting cells CD11c+ dendritic cells (Ā: 1.39% vs. 0.87%), CD11b+ macrophages (Ā: 3.22% vs. 0.81%) and gamma delta T (γδ T) cells (Ā: 5.56% vs. 1.35%) were increased. In adult Ames dwarf mice, adaptive immune cells, such as IL-17 producing CD4+ T helper (Th17) cells (Ā: 8.3% vs. 4.7%) were augmented. In the MLNs of Ames dwarf mice, the antigen presenting and adaptive immune cells also altered when compared to WT mice, such as a decrease of T-regulatory (Treg) cells in juvenile Ames mice (Ā: 7.7% vs.10.5%), but an increase of Th17 cells (Ā: 0.627% vs.0.093%). Taken together, these data suggest that somatotropic signaling deficiency influences colon development and intestinal immunity.

## Introduction

Ames dwarf mice possess a spontaneous *Prophet of Pituitary Factor 1* (*Prop1*) loss-of-function mutation. The mutation of the Prop1 gene results in the lack of differentiation of endocrine cell lineages (somatotrophs, lactotrophs and thyrotrophs) in the anterior pituitary. Therefore, Ames dwarf mice are deficient in growth hormone (GH) and insulin-like growth factor 1 (IGF-1), thyroid-stimulating hormone (TSH), the thyroid hormones (THs), and prolactin (PRL) [1]. Particularly important is the deficiency of GH and IGF-1 signaling (collectively referred to as somatotropic signaling) [2], which is believed to be the principle force behind the approximately 50% increase in longevity observed in Ames dwarf mice (depending on sex and diet) [3]. Mechanisms that are responsible for the longevity of Ames dwarf mice may include improved antioxidant defense, enhanced insulin sensitivity and reduced insulin levels, reduced inflammation and cell senescence, and greater stress resistance [4]. However, it is unclear how the somatotropic signaling (GH/IGF-1) defect affects the colon development and intestinal immunity in these mice.

Beyond the main role of promoting linear growth and metabolism, GH has important effects on the immune system. For instance, GH can interact with B and T lymphocytes [5], promote thymic growth and T cell development, improve T cell function, and enhance the immune response [6,7], playing an important role in homeostasis of immune system. High endogenous GH levels inhibit specific antibody (Ab) production and peripheral T cell populations, but do not impact peripheral B cell number, Th2 cell population, and the production of interleukin-4 (IL-4) and IFN-γ [8]. GH and IGF-I are involved in regulation of antioxidative stress and in Ames dwarf mice, GH and IGF deficiency may cause oxidative stress [9]. In addition, Ames dwarf mice have increased level of adiponectin and reduced expression of interleukin-6 (IL-6) and tumor necrosis factor-alpha (TNF-α) [10,11]. Ames dwarf mice with pituitary grafts at 21 days showed increased lymphocytes in the spleen and thymus, splenic natural killer (NK) cell activity and peripheral white blood cells [12].

The gastrointestinal tract is exposed to a large variety of food antigens and resided with a huge amount of commensal bacteria, playing an important role in human health [13]. The intestinal tract is also an active player of the local and systemic immune system, participating in both the innate and adaptive immune responses [14]. Impairment of intestinal epithelial barrier [15] and environmental factors (like gut microbiota, antibiotics and diet) [16] may cause immunological imbalance and influence distinct arms of the immune response. Impaired gastrointestinal function contributes to aging [18], and the hypothalamic-pituitary-adrenal axis modulates the gut microbiota in mice [19].

In the intestine, GH regulates enteroendocrine cell secretion, calcium absorption, and intestinal amino acid and ion transport. GH also functions in the growth of intestinal mucosa and increase the proliferative activity of intestinal stem cells [20], which is critical to the gut mucosal integrity and immunity. Therefore, the somatotropic signaling-deficient mice provide a novel model for investigation of the role of GH in intestinal development and immunity [21,22]. To date, however, the mucosal development and intestinal immunity of the Ames dwarf mice remains unclear. In this current study, we observed the colon structure and the inflammatory/immune cells in the colon lamina propria (cLP) and MLNs in 2 months (juvenile) and 6 months (adult) old Ames dwarf and age-matched WT control mice. The results demonstrated abnormal cryptic development and alterations in the innate immune cells (such as neutrophils, monocytes, eosinophils, γδ T cells, CD11c+ DCs and CD11b+ macrophages) and adaptive immune cells (i.e., B220+ B cells, CD4+ Th1, Th17 and Treg helper cells). This study represents the first observation of intestinal immunity in Ames dwarf mice.

## RESULTS

### Dwarf mice demonstrate age-related alterations in colon development and MLN weight

**Figure 1:**
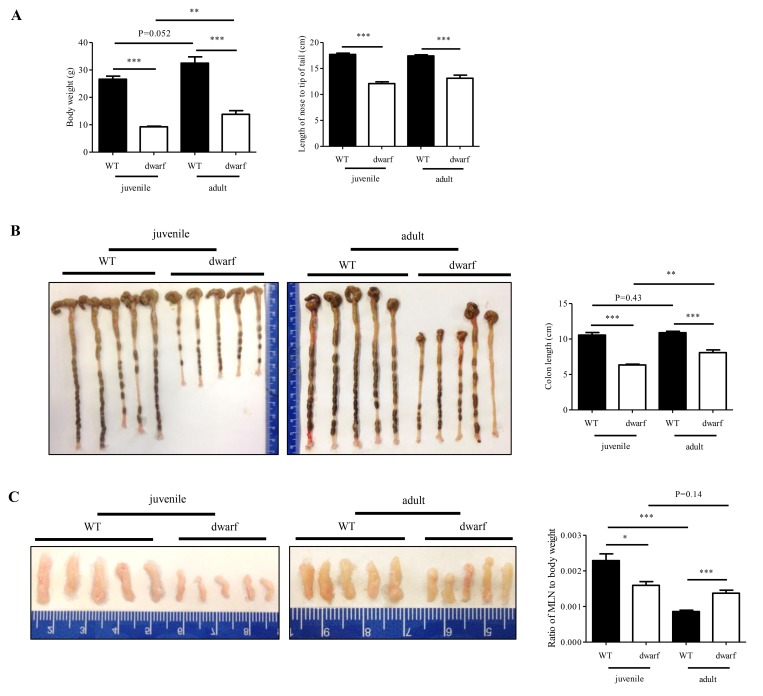
**Age-related alterations in body weight, colon length and MLN weight of dwarf mice.** (**A**) Body weight (*left panel*) and body length (*right panel*) in juvenile and adult mice. (**B**) Colon length in juvenile (*left panel*) and adult (*middle panel*) mice. *Right panel*, average length of five colons. (**C**) MLN size in juvenile (*left panel*) and adult (*middle panel*) mice. *Right panel*, ratio of MLN weight/body weight in five mice. *, P < 0.05; **, P < 0.01 and ***, P < 0.001 compared to WT.

Juvenile and adult Ames dwarf mice are smaller in body weight and body length than age-matched WT mice (Figure 1A), which is consistent with literature report [23]. However, the colon length and MLNs weight of Ames dwarf mice showed age-related changes. The colons of juvenile Ames dwarf mice were approximately 40% shorter than those of juvenile WT mice, but the difference was reduced to about 24% in adult Ames dwarf mice (Figure 1B). The colon length of WT mice was not changed from juvenile to adult. These data indicate that colon development was completed by two months old in WT mice, but not in Ames dwarf mice. A great age-related change was observed in MLNs. As shown in Figure 1C, MLNs were small in juvenile Ames dwarf mice with a ratio of MLNs over body weight at an average of 0.00159 (n=5) vs. 0.00229 in juvenile WT mice. In adult Ames dwarf mice, MLNs were much larger with a ratio of MLNs to body weight at an average of 0.00137 (n=5) vs. 0.00086 in adult WT mice. These gross data indicate age-related changes in colon length and MLNs size in Ames dwarf mice.

### Shorter colonic crypts in Ames dwarf mice

**Figure 2:**
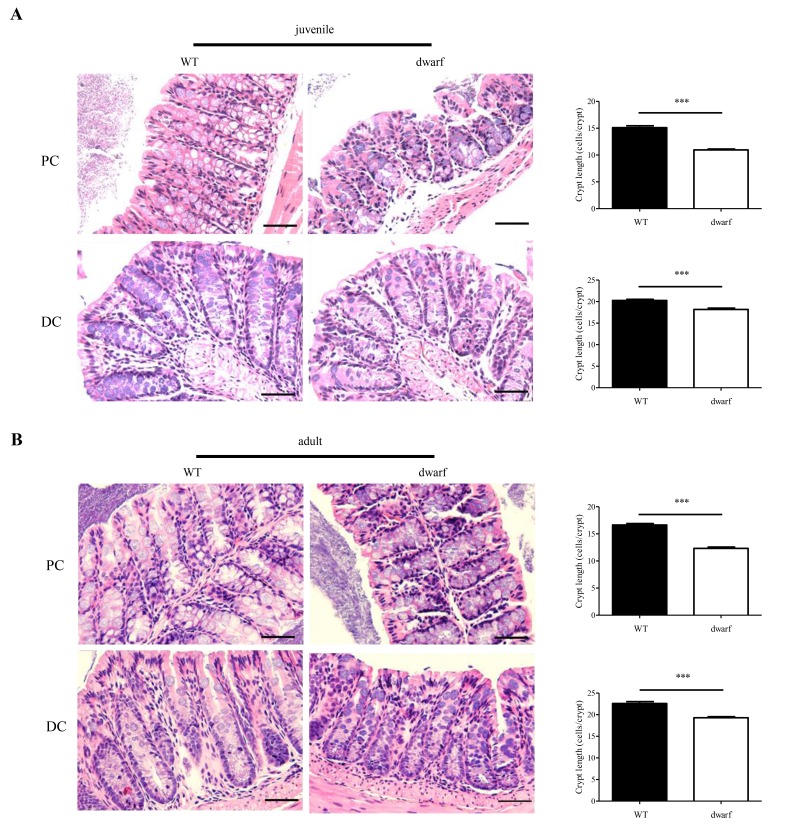
**Colonic crypt length in dwarf mice**. H&E staining sections of proximal colon (PC) and distal colon (DC) were used for evaluation of colonic crypt length. (**A**) Juvenile mice. *Left panel*, images of colonic crypts; *right panel*, cryptic cell number from 40 integrated crypts per mouse. (**B**) Adult mice. *Left panel*, images of colonic crypts; *right panel*, cryptic cell number from 40 integrated crypts per mouse. N=5; ***, P < 0.001 compared to WT. Scare bar: 50μm.

The gross changes of colon length in Ames dwarf mice encouraged an evaluation of colon histology. Colonic crypts which are composed of epithelial cells contribute to host-microbial homeostasis, antimicrobial defense and modulation of immune response [22]. The length of a crypt is defined by cell number in the crypt and varies in different parts of colon. We evaluated the cell number in the crypts of proximal colons (PC) and distal colons (DC), respectively. The results showed that the colonic crypts in PC and DC were shorter in Ames dwarf mice than in WT mice. In juvenile mice, the proximal colon had 15±3.5 cells/crypt in WT mice, but only 11±2.3 cells/crypt in Ames dwarf mice (n=30, P < 0.001); the distal colon had 20±2.7 cells/crypt in WT mice and 18±2.8 cells/crypt in dwarf mice (n=30, P < 0.001) (Figure 2A). In adult mice, the proximal colon had 16±2.7 cells/crypt in WT mice, but only 12±2.9 cells/crypt in dwarf mice (n=30, P < 0.001); the distal colon had 22±4.4 cells/crypt in WT mice and 19±3.1 cells/crypt in dwarf mice (n=30, P < 0.001) (Figure 2B). These data indicate that the Ames dwarf mice have developmental abnormalities in colonic crypts.

### Alterations of inflammatory and immune cells in colonic lamina propria (cLP) of Ames dwarf mice

**Figure 3:**
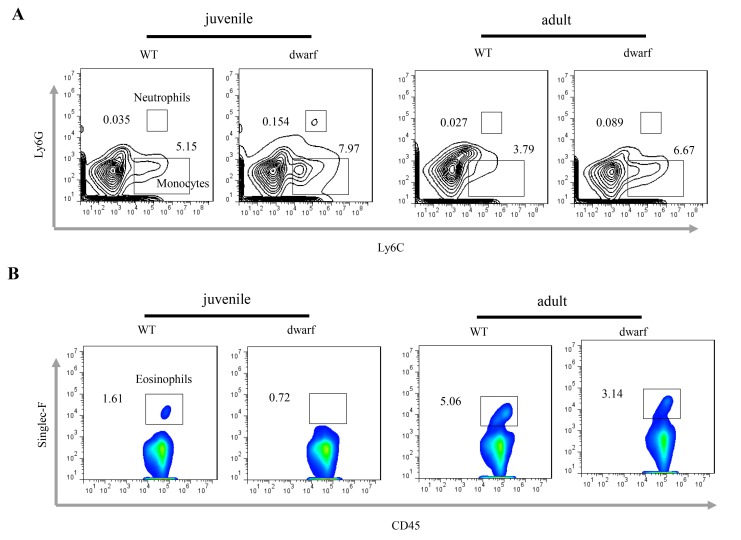
**Inflammatory cells in the cLP of dwarf mice.** (**A**) Neutrophils and monocytes in juvenile and adult mice. (**B**) Eosinophils in juvenile and adult mice. Data (percentage) in images indicate the results from a pool of cLP cells from 5 mice.

Defects in colon development may alter the intestinal immune cells. We observed inflammatory cells in colonic lamina propria (cLP). The results showed that the mean percentage of Ly6C+ Ly6G+ neutrophils (Ā: 0.154 vs. 0.035 in juvenile and 0.089 vs. 0.027 in adult) and Ly6C+ Ly6G- monocyte (Ā: 7.97 vs. 5.15 in juvenile and 6.67 vs. 3.79 in adult) was noticeably increased in the cLP of juvenile and adult Ames dwarf mice when compared to WT mice at the same age (Figure 3A), indicating mild inflammation in the colon of Ames mice. Interestingly, the percentage of eosinophils, a type of blood cell involved in allergic reaction and parasitic infection, was decreased in juvenile (Ā: 0.72 vs. 1.61) and adult (Ā: 3.14 vs. 5.06) Ames dwarf mice (Figure 3B).

**Figure 4:**
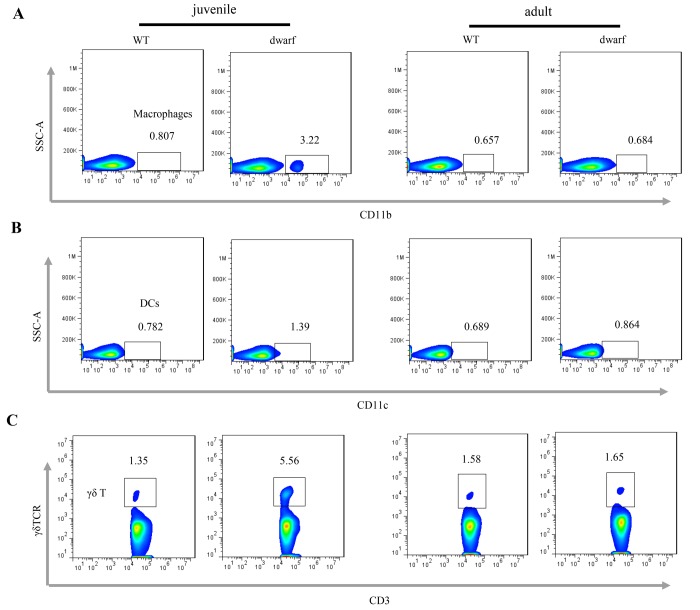
**Antigen presenting cells in the cLP of dwarf mice.** (**A**) CD11b+ macrophage, (**B**) CD11c+ dendritic cells and (**C**) γδ T cells in the cLP of juvenile and adult mice. Data (percentage) in images indicate the results from a pool of cLP cells from 5 mice.

**Figure 5:**
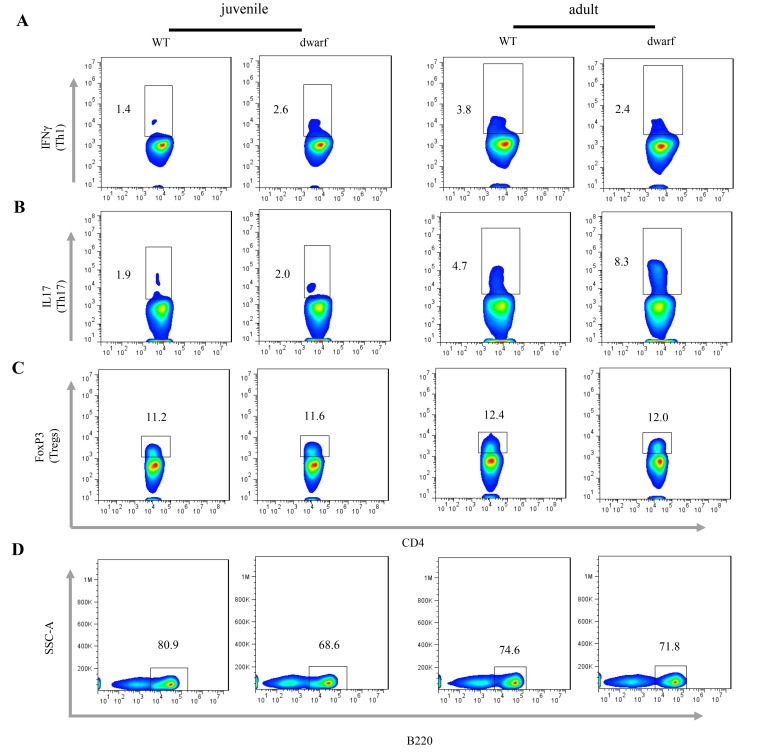
**Adaptive immune cells in the cLP of dwarf mice.** (**A**) Th1 cells, (**B**) Th17 cells, (**C**) Treg cells and D) B cells in the cLP of juvenile and adult mice. Data (percentage) in images indicate the results from a pool of cLP cells from 5 mice.

We further evaluated antigen presenting cells (APCs) in the cLP of Ames dwarf mice, including CD11b+ macrophages, CD11c+ dendritic cells (DCs) and γδ T cells. The results showed that the mean percentage of CD11b+ macrophages (Ā: 3.22 vs. 0.81), CD11c+ DCs (Ā: 1.39 vs. 0.78) and γδ T cells (Ā: 5.56 vs. 1.35), was markedly increased in juvenile dwarf mice when compared to WT counterparts (Figure 4A-C), but in adult dwarf mice only DCs (Ā: 0.864 vs. 0.689) were slightly increased (Figure 4B). The increased APCs encouraged us to investigate the adaptive immune cells in cLP. The results showed that it was mainly the Th1 helper cells (Ā: 2.6 vs. 1.4) that increased in juvenile Ames dwarf mice (Figure 5A), but was mainly the Th17 helper cells (Ā: 8.3 vs. 4.7) in adult dwarf mice (Figure 5B**)**. Treg cells were not noticeably changed in both juvenile (Ā: 11.2 vs. 11.6) and adult (Ā: 12.4 vs. 12.0) Ames dwarf mice (Figure 5C**)**. In addition, we observed a decrease of B220+ B cells (Ā: 68.6 vs. 80.9) in juvenile dwarf mice, but not in adult dwarf mice (Figure 5D). Together these data indicate the changes of intestinal immunity in Ames dwarf mice.

### Changes of immune cells in mesenteric lymph nodes of Ames dwarf mice

Considering the changes in the size of the MLNs and in immune cells of cLP, we further investigated the immune cells in MLNs. As summarized in Table 1, the percentage of CD11c+ DCs was higher in both juvenile (Ā: 0.581 vs. 0.469) and adult (Ā: 0.57 vs. 0.453) dwarf mice when compared to WT mice. However, the CD11b+ macrophages showed age-related variations. The CD11b+ macrophages were less in the juvenile dwarf mice (Ā: 0.46 vs. 1.38), but more in adult dwarf mice (Ā: 3.47 vs. 1.35) compared with WT mice. The percentage of γδ T cells was slightly higher at (Ā: 0.903 vs. 0.817) in juvenile dwarf mice, but is lower at (Ā: 1.45 vs. 1.76) in the adult dwarf mice. Furthermore, we observed less Th1 cells in juvenile (Ā: 0.171 vs. 0.373) and adult (Ā: 1.29 vs.2.15) and B cells in juvenile (Ā: 33.4 vs. 38.3) and adult (Ā: 32.4 vs. 37.2). However, the percentage of Th17 cells were greatly increased at (Ā: 0.627 vs. 0.093) in juvenile dwarf mice while no differences were observed in adult dwarf mice. These data indicate age-related changes of immune cells in MLNs.

**Table 1:** Innate and adaptive immune cells in MLNs of juvenile and adult Ames dwarf mice. Values are mean percentage (Ā).

**Cells**	**juvenile**		**adult**		**Gating**
	**WT (%)**	**dwarf (%)**	**WT (%)**	**dwarf (%)**	
Tregs	10.5	7.7	10.9	11.5	FoxP3+ (FSC-SSClow 7AAD- CD4+)
Th1	0.373	0.171	2.15	1.29	IFNγ+ (FSC-SSClow 7AAD- CD4+)
Th17	0.093	0.627	0.74	0.63	IL17+ (FSC-SSClow 7AAD- CD4+)
γδ T	0.817	0.903	1.76	1.45	γδTCR+ (FSC-SSClow 7AAD- CD3+)
Macrophages	1.38	0.46	1.35	3.47	CD11b+ (FSC-SSClow 7AAD- CD45+ )
Dendritic cells	0.469	0.581	0.453	0.571	CD11c+ (FSC-SSClow 7AAD- CD45+ )
B cells	38.8	33.4	37.2	32.4	B220+ (FSC-SSClow 7AAD- CD45+)

## DISCUSSION

The present study investigated the colon development and immune cells in the cLP and MLNs of Ames dwarf mice with WT mice at the same age as a control. Ames dwarf mice are GH-deficient due to a mutation in a pituitary-specific, paired-like homeodomain transcription factor gene, *Prop1*. The growth and maturation of dwarf mice are delayed and we assayed the immune cells at the ages of 2 months and six months old, respectively. Herein we termed the mice at 2 months as juvenile and mice at 6 months as adult.

Ames dwarf mice were 40-50% less of body weight compared to their WT counterparts at the same ages, but the ratio of MLNs to body weight was dramatically changed with age. In juvenile dwarf mice, the MLN to body weight was smaller in Ames dwarf mice than in WT mice, but in adult dwarf mice this ratio was larger, indicating an active immune response in the intestine of adult dwarf mice. The colons were significantly shorter in juvenile and adult dwarf mice than in WT mice, but when compared between the juvenile and adult Ames mice, the colons of adult dwarf mice were longer, indicating the delayed maturation of colon in the Ames dwarf mice. More significantly, the dwarf mice had shorter crypt length in both the proximal colon and distal colon when compared with WT counterparts, indicating that the deficiency of somatotropic signaling (GH/ IGF-1), together with TSH, THs and prolactin signaling, may affect the growth and development of the colonic crypts. Dwarf mice have a delay in puberty, and it is largely due to lack of thyroid hormone [24]. Herein we found the developmental defects of the colon in Ames dwarf mice, but it is unknown how the deficiency of somatotropic signaling affects the development of the colon. Further study is warranted.

Intestinal epithelial cells (IECs) could produce various types of cytokines and chemokines, which function as immunoregulatory signals for directing immune cell response against foreign antigens [25]. The defect of colonic crypts in Ames dwarf mice may dysfunction the immunoregulatory signaling and lead to alterations of the innate and adaptive immune response. In addition, IECs are a single layer of cells that separate the host from gut contents that are enriched with pathogens and commensal bacteria. The intestinal microbiota plays a crucial role in the development of local and systemic immunity [26,27]. For example, colonization of the small intestine of mice with segmented filamentous bacteria could induce Th17 cells response and antimicrobial defense [16] and increase the number of Treg cells in the small intestine and colon [28]. Colonization of animals with the gut microorganism *Bacteroides fragilis* directs the cellular and physical maturation of the developing immune system, promoting Th1/Th2 balance [29]. Additionally, microbiota could also drive the expansion of B and T cells in Peyer’s patches and mesenteric lymph nodes [30], promoting IgA secretion [31]. Therefore, we further investigated the inflammatory and immune cells in the cLP and MLNs.

Neutrophils and monocytes are the first-responders of inflammatory cells that migrate towards the site of inflammation, and play specific and nonspecific defensive functions. In both juvenile and adult dwarf mice, neutrophils and monocytes were increased in the cLP when compared to WT mice, indicating presence of mild inflammation in the colon of dwarf mice. Interestingly, the eosinophils were decreased in juvenile and adults dwarf mice compared to WT mice. Eosinophils are innate immune cells that function in regulation of inflammation, epithelial barrier, tissue remodeling and bridging of innate and adaptive immunity [32]. Eosinophil peroxidase forms reactive oxygen species and reactive nitrogen intermediates that can promote oxidative stress and killing of microbial pathogens. The decreased eosinophils in the colon suggest the dysfunction of intestinal barrier and inflammation.

The increased inflammatory cells in cLP were accompanied with the elevation of antigen presenting cells in juvenile and dwarf mice, including CD11b+ macrophage, CD11c+ DCs and γδ T cells. The γδ T cells are innate-like lymphoid cells and can function in the resolution of infection by multiple ways, such as TCR-MHCII independent antigen presentation and recruitment of effector cells like neutrophils and macrophages, playing an important role in the immune surveillance [33]. The increase of these cell populations suggests enhanced pathogen exposure of the colon in dwarf mice with defects in cryptic development, which is consistent with the presence of mild inflammation as indicated by increased inflammatory cells. However, it is noteworthy that hematopoietic stem cells (HSCs) are increased in juvenile and adult Ames dwarf mice [34]. Whether the changes of HSC in bone marrow influence the inflammatory cells in cLP of Ames dwarf mice is warranted for further study.

Furthermore, consistent with increased antigen presenting cells, adaptive immune cells were also increased in dwarf mice although variations occurred in juvenile and adult dwarf mice. Th1 cells were increased in juvenile dwarf mice, but slightly decreased in adult dwarf mice. Th17 cells were not altered in juvenile dwarf mice, but markedly increased in adult dwarf mice. Literature data show the pivotal beneficial role of IL-17A for the integrity of the intestinal epithelial barrier, and mice with deficiency of IL-17 display a broad vulnerability to various infectious pathogens [35]. The increased Th17 cells in adult dwarf mice may play a protective role, contributing to host defense and barrier integrity in the dwarf mice.

The abnormal size of MLNs (ratio of MLN/body weight) indicates increased inflammatory/immune responses. Thus we investigated the antigen presenting cells and adaptive immune cells in MLNs. Results showed a decrease of CD11b+ macrophages, Th1 and Treg cells, but a great increase of Th17 cells in juvenile dwarf mice. In the adult dwarf mice, CD11b+ macrophages were elevated about 3 fold. These results indicate an active immune response of MLNs to intestinal pathogens.

Colonization of gut microbiota in early life plays an instrumental role in the development and education of the host immune system [36], and alterations of intestinal commensals have profound effects on the structural and functional development of the immune system, such as T cell response [37]. This study found that in Ames dwarf mice, the deficiency of GH, PRL and TSH led to defects of colonic epithelial proliferation and cryptic development although the underlying mechanism is unclear yet. As an important immune tissue and barrier, this may cause abnormal immune response, commensal colonization and bacterial infiltration. Our results in inflammatory and immune cells in the cLP and MLNs support this hypothesis and are of important significance. The Ames dwarf mice may be a great novel model to understand intestinal homeostasis, commensal colonization and immune development.

## MATERIALS AND METHODS

### Animals

Male Ames dwarf mice were produced in our closed breeding colony at Southern Illinois University School of Medicine (SIUSOM) by breeding homozygous mutant males (*Prop1^-/-^*) with heterozygous females (*Prop1^+/-^*). All breeding pairs avoided brother x sister mating to ensure genetic diversity. Heterozygous normal (WT) animals were used as controls for the homozygous mutant dwarfs. Animals entered the study at 2 and 6 months of age.

All animals were housed and bred in a temperature-controlled (22°C) room with a daily photoperiod of 12 hr:12 hr as light:dark. Rodent food (Formulab Laboratory Diet, PMI Nutrition International, Inc., St. Louis, MO) and tap water were supplied *ad libitum*. All animal procedures were approved by the SIUSOM Animal Care and Use Committee.

### H&E staining

Hematoxylin and Eosin (H&E) staining was performed using a standard laboratory protocol. In short, tissues were fixed overnight in 10% formaldehyde for paraffin embedding. The embedded samples were sectioned at 4µm thickness, followed by staining with H&E staining. Sections were examined using 400× magnification, all images were captured on a microscope (Olympus, Japan). Crypt length was defined as cell number per crypt by counting 30 integrated crypts per mouse.

### Cell isolation

Colonic lamina propria cells were isolated following an established protocol [38]. Briefly, after flushing the intestinal contents with cold PBS, the colons from 5 mice were opened longitudinally, pooled and cut into small pieces of ∼1 cm in length, followed by incubation twice for 20 min in PBS supplemented with 2% FBS, 5 mM EDTA and 1mM D,L-dithiothreitol (DTT; American Bioanalytical). The tissues were then cut into 1 mm pieces and further incubated in HBSS (Hank’s Balanced Salt Solution) in the presence of 0.5 mg/ml collagenase D (Roche), 0.5 mg/ml dispase II (Roche) and 100 U DNase I (Sigma) for two consecutive 20 min at 37˚C. After digestion, cells were passed through a 40μm nylon cell strainer (Fisher Scientific) and then recovered by Percoll gradient centrifugation at 2500 rpm for 20 min. Leukocytes were recovered at the interface of 40%/80% Percoll, then washed and kept in cell staining buffer (BioLegend). For cells isolated from the MLN, cut the tissue into pieces, then pass the tissue through a 40μm nylon cell strainer (Fisher Scientific) by using a plunger, the cells were collected, washed and then kept in cell staining buffer (BioLegend).

### Cell stimulation and staining

For flow cytometry analysis, isolated cells from the cLP and MLNs were pre-incubated with an Fc receptor-blocking mAb (CD16/32; 2.4G2) for 10 minutes at 4 °C, then incubated with saturating amounts of FITC-, PE- and APC-conjugated mAbs for 30 minutes at 4 °C. To assess intracellular IL-17A, IL-22 and IFNγ, cells were stimulated for 5hr in medium RPMI 1640 containing 10% FBS, 50 ng/ml Phorbol 12-Myristate 13-Acetate (PMA; SigmaAldrich, St. Louis, MO) and 500 ng/ml ionomycin (Sigma-Aldrich) in the presence of GolgiStop (BD Pharmingen). After surface staining, stimulated cells were fixed with fixation buffer (BioLegend) and permeabilized with permeabilization wash buffer (BioLegend), followed by intracellular cytokine staining. To detect intracellular Foxp3, a BioLegend true-nuclear transcription factor buffer set was used for fixation and permeabilization of the cells.

### Antibodies and flow cytometry

Abs for FACS analysis, Fc γ receptor-blocking mAb (CD16/32; 2.4G2), FITC-conjugated-conjugated mAbs: CD3 (145-2C11), IFN-γ (XMG1.2), CD45 (30-F11); PE-conjugated mAbs: TCRγδ (GL-3), CD4 (GK1.5), Ly6G (1A8), CD11c (N418), B220 (RA3-6B2), Siglec-F(E50-2440); APC-conjugated mAbs: IL17 (TC11-18H10.1), CD11b (M1/70), and Ly6C (HK1.4) were used. The florescence of the cells was analyzed using an Accuri C6 flow cytometer (Accuri, Ann Arbor, MI, USA) and the data were analyzed with FlowJo software (TreeStar, San Carlos, CA). Cell gating strategies are shown in Figure S1. Briefly, after excluding the debris and doublets, cells were gated on 7AAD-live cells, then CD45+ leukocytes. All the percentages of immune cells in the paper were the mean percentage (n=5) indicated as Ā.

### Statistical analyses

Statistical analyses were carried out with Prism 4 (Graph Pad software, CA). Variance test, Student t test, or one-way ANOVA test, as appropriate, were used to compare the difference between WT and Ames dwarf mice with p < 0.05 as statistical significance. *, P < 0.05, **, P < 0.01 and ***, P < 0.001 compared with WT.

## Supplementary Material

Supplementary File
