# Supplementary material for: Intestinal immunity in hypopituitary dwarf mice: effects of age
Source: Aging (Albany NY). 2018 Mar 2;10(3):358–70. doi: 10.18632/aging.101393 (PMC5892686; doi:10.18632/aging.101393)
Supplement: Supplementary File [file aging-10-101393-s001.pdf]

SUPPLEMENTARY MATERIAL

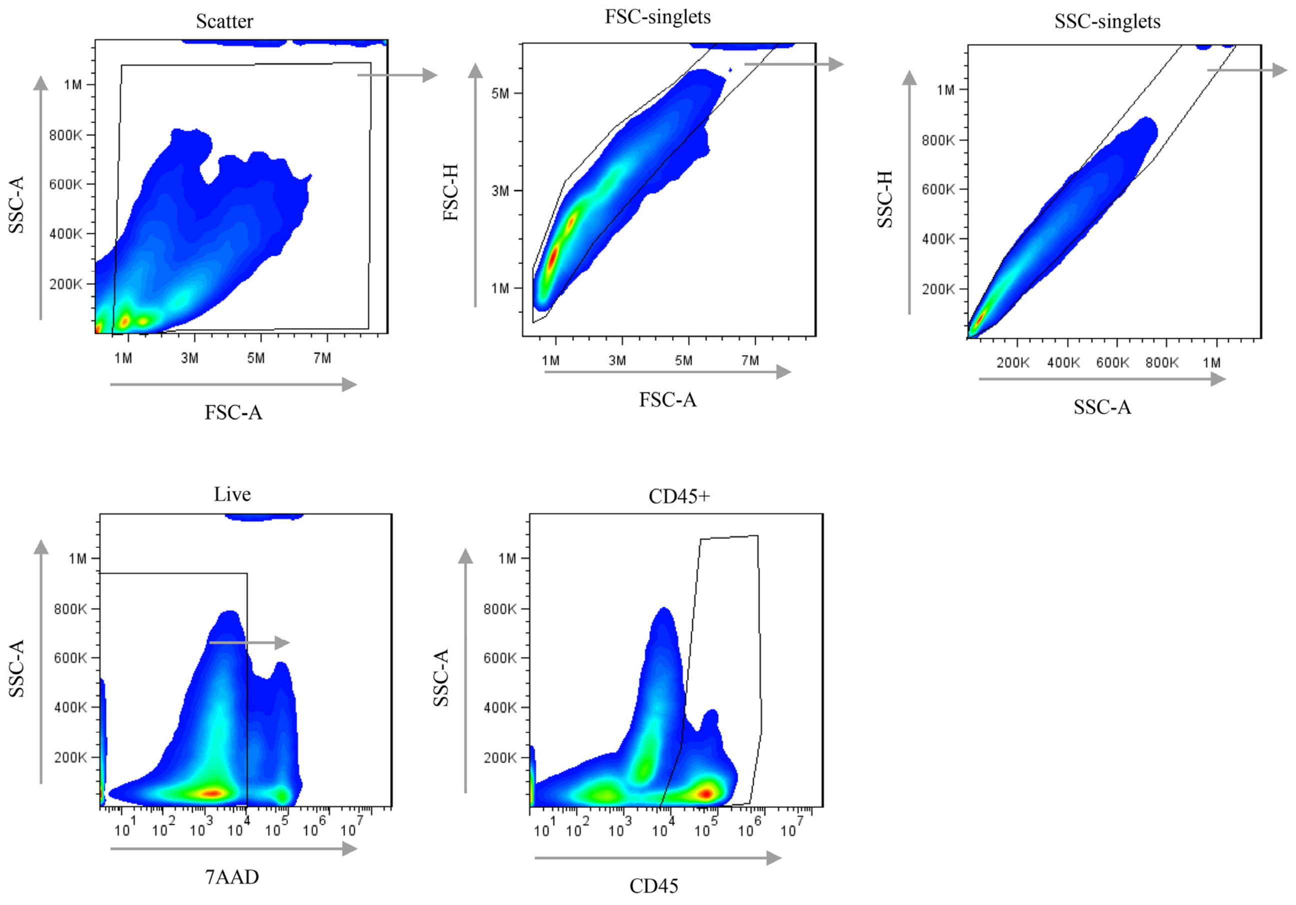

Supplemental Figure 1. Gating strategies in the analysis of leukocytes from cLP. From left to right: Scatter gates to exclude debris, singlets gates to exclude doublets, live gate to exclude 7AAD + dead cells, and then gate on CD45+ leukocytes. FSC, forward light scatter; SSC, side light scatter.
